# The Pedicled Anterolateral Thigh Flap for Phalloplasty: The Athenian Experience

**DOI:** 10.7759/cureus.112731

**Published:** 2026-07-15

**Authors:** Spyridon P Koliopoulos, Eleni Markaki, Dimosthenis Chrysikos, George Tsakotos, Nikolaos A. Papadopulos

**Affiliations:** 1 Department of Plastic Surgery, Eugenidio University Hospital, Athens, GRC; 2 Department of Anatomy, Medical School, National and Kapodistrian University of Athens, Athens, GRC; 3 Department of Maxillofacial Surgery, Attikon University Hospital, Athens, GRC

**Keywords:** anterolateral thigh flap, female-to-male transgender surgery, gender-affirming surgery, nerve coaptation, pedicled flap, penile reconstruction, phalloplasty, reconstructive microsurgery

## Abstract

In terms of preoperative planning and surgical procedure, phallus reconstruction (phalloplasty) in transgender men is an extremely difficult treatment, and numerous operating techniques have been previously documented. Here, we present the first reported case in Greece of a phalloplasty using a pedicled, aesthetically innervated anterolateral thigh (ALT) flap while maintaining the neourethra, which was constructed previously as part of metoidioplasty gender-affirming surgery. Both procedures were performed by the same senior surgeon in a 32-year-old patient. There were no intraoperative complications, and the operative duration remained within the expected range. When used as a salvage technique in a preoperated case with altered vascular anatomy, the advantages of a pedicled transposition over a free flap transplant are obvious and need to be addressed. Alone, the lack of microvascular anastomoses has the advantages of a shorter operation time and lack of potential post-operative complications. In conclusion, we view our phalloplasty procedure as a suitable substitute for existing treatments in some patients with regard to operating time, effort time, complications, donor-site morbidity, and cosmetic outcome.

## Introduction

One of the most difficult reconstructive surgical procedures is phalloplasty, which is carried out on patients who need penile repair because of trauma, oncologic excision, congenital abnormalities, or as part of gender-affirming surgery. The construction of a neophallus with good cosmetic appearance, preservation or restoration of sensation, and maintenance of urine function are the main objectives of phalloplasty [1-3].

Radial forearm free flaps, abdominal flaps, free osteofasciocutaneous fibula flaps, and thigh-based flaps are among the reconstructive procedures that have been reported. Although the radial forearm flap is the gold standard, the unsightly donor-site scar and complications such as flap necrosis, urethral fistula, and strictures continue to be its principal disadvantages. As a general rule, the quality and placement of the perforators are crucial for a successful repair, much like in all perforator flaps. Traditionally, preoperative perforator mapping is often carried out using indocyanine green angiography, colour Doppler ultrasonography, and standard Doppler ultrasound, as in our case [4-7].

The complete anatomy of the anterolateral thigh (ALT) flap has drawn more attention among these methods because of its advantageous features, which include a broad skin paddle, dependable vascular architecture, and comparatively minimal donor-site morbidity. This method is a desirable alternative to forearm-based repair since the donor-site scar can frequently be hidden [8,9].

Simplified phalloplasty methods that do not require neourethra elongation may be suitable for certain patients, as in this case. Avoiding urethral extension permits the development of a neophallus with good aesthetic and sensory results while lowering the likelihood of common complications such as urethral fistula and stricture. The possibility of sensory recovery may also be enhanced by neurorrhaphy between the clitoris dorsal nerves and the flap’s sensory nerve [5,10].

Based on perforators from the descending branch of the lateral circumflex femoral artery, the ALT flap is a fasciocutaneous flap that offers a constant pedicle of sufficient length for pedicled transfer and a dependable vascular supply. These perforators, which might be musculocutaneous or septocutaneous, are usually seen along the line that joins the lateral patella to the anterior superior iliac spine. Accompanying venae comitantes guarantee venous drainage. The lateral femoral cutaneous nerve, which provides sensory innervation, can be included into the flap to provide neurorrhaphy for possible sensory restoration, as in this instance through coaptation to the dorsal nerve of the clitoris. Although flap thickness may require subsequent contouring, the donor location affords a concealable scar and comparatively minimal morbidity [11,12].

In this case report, we describe the anatomy of the flap, the surgical approach, and the postoperative results of a phalloplasty employing a pedicled ALT flap with preservation of the native urethra and sensory nerve coaptation.

## Case presentation

**Figure 1:**
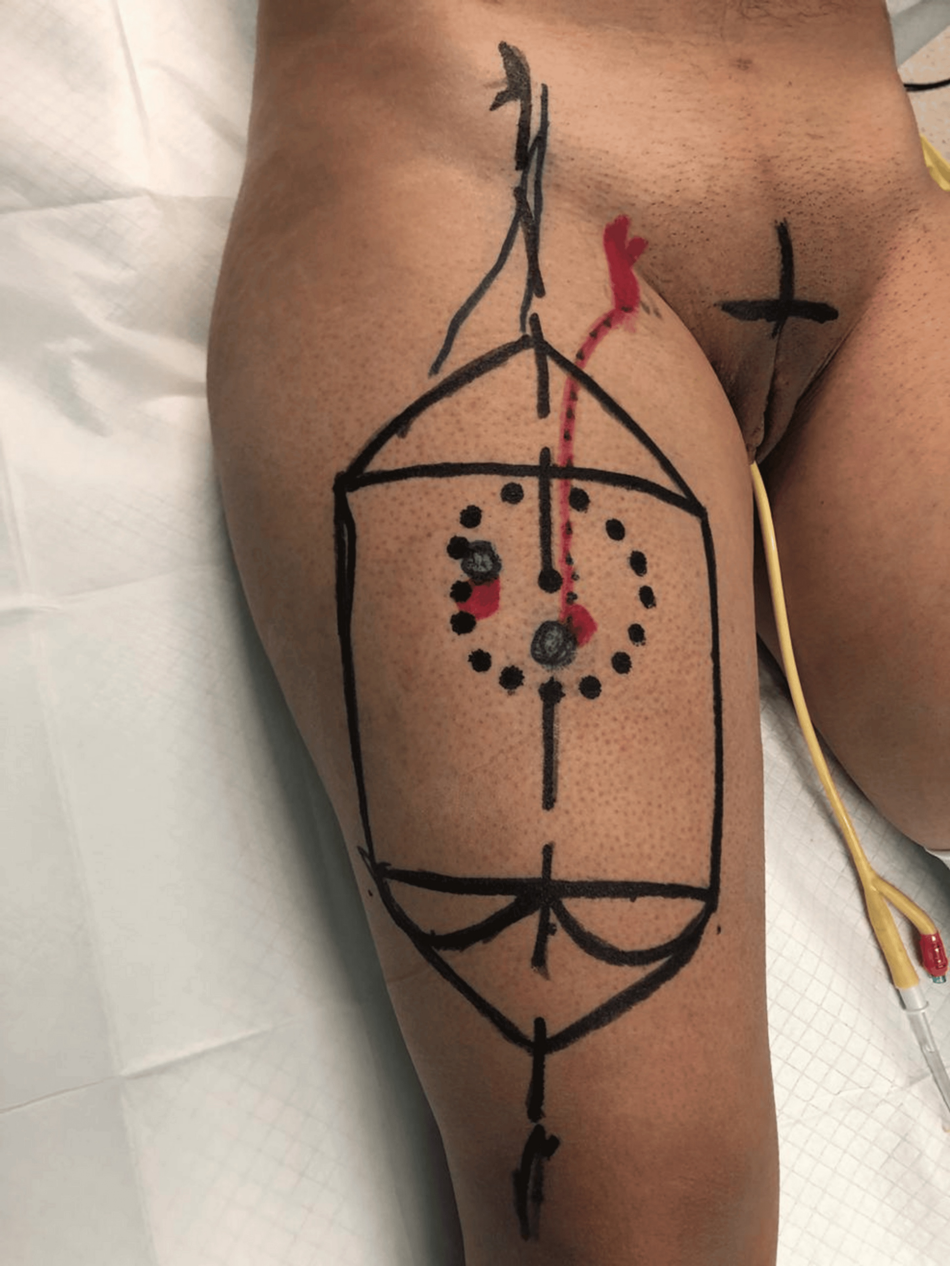
Design of the anterolateral thigh (ALT) flap and marking of the perforating vessels using Doppler sonography. The four large dots, located near the dotted line, depict the perforator vessels of the flap. The rectangle with the triangles at the top and the bottom represents the future phallus.

General anaesthesia was administered while the patient was in a supine position, and preoperative markings were made. A Foley catheter was placed, and both the donor and recipient sites on the anterior thigh were sterilely prepped and covered. The anterolateral part of the thigh was marked in order to design a skin paddle that was rectangular with elliptical edges and measured about 15 × 14 cm. With the use of portable Doppler ultrasound, the perforator vessels were marked. The longitudinal line between the anterior superior iliac spine and the superolateral edge of the patella was marked by strong perforating vessels, originating from the descending branch of the lateral circumflex femoral artery (Figure 1).

The flap’s purpose was to supply enough tissue for the construction of the phallus. To guarantee sufficient vascular supply, special consideration was given to the direction of the skin paddle.

**Figure 2:**
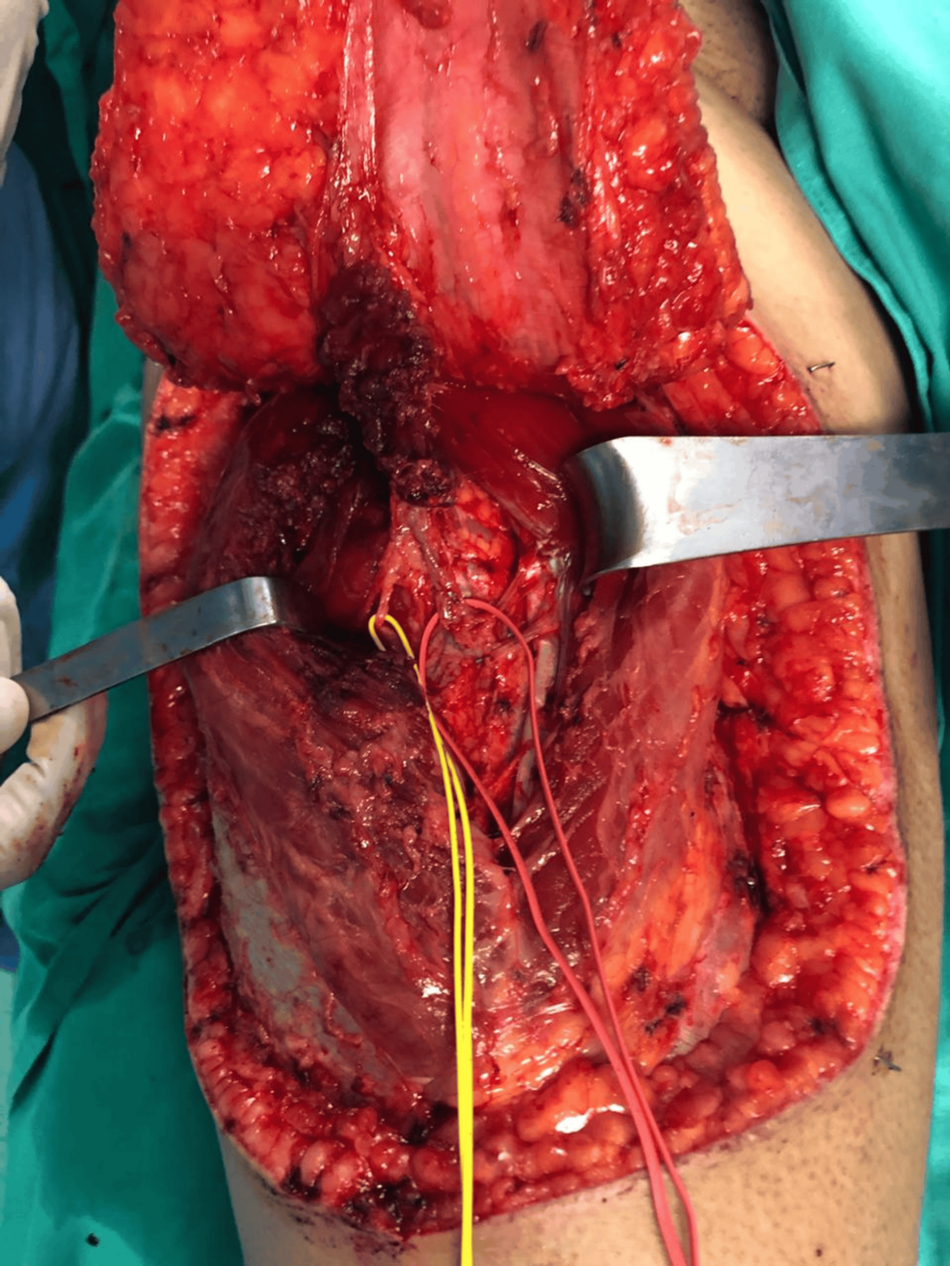
Identification of the innervation (yellow) and vascularisation (red) of the flap.

**Figure 3:**
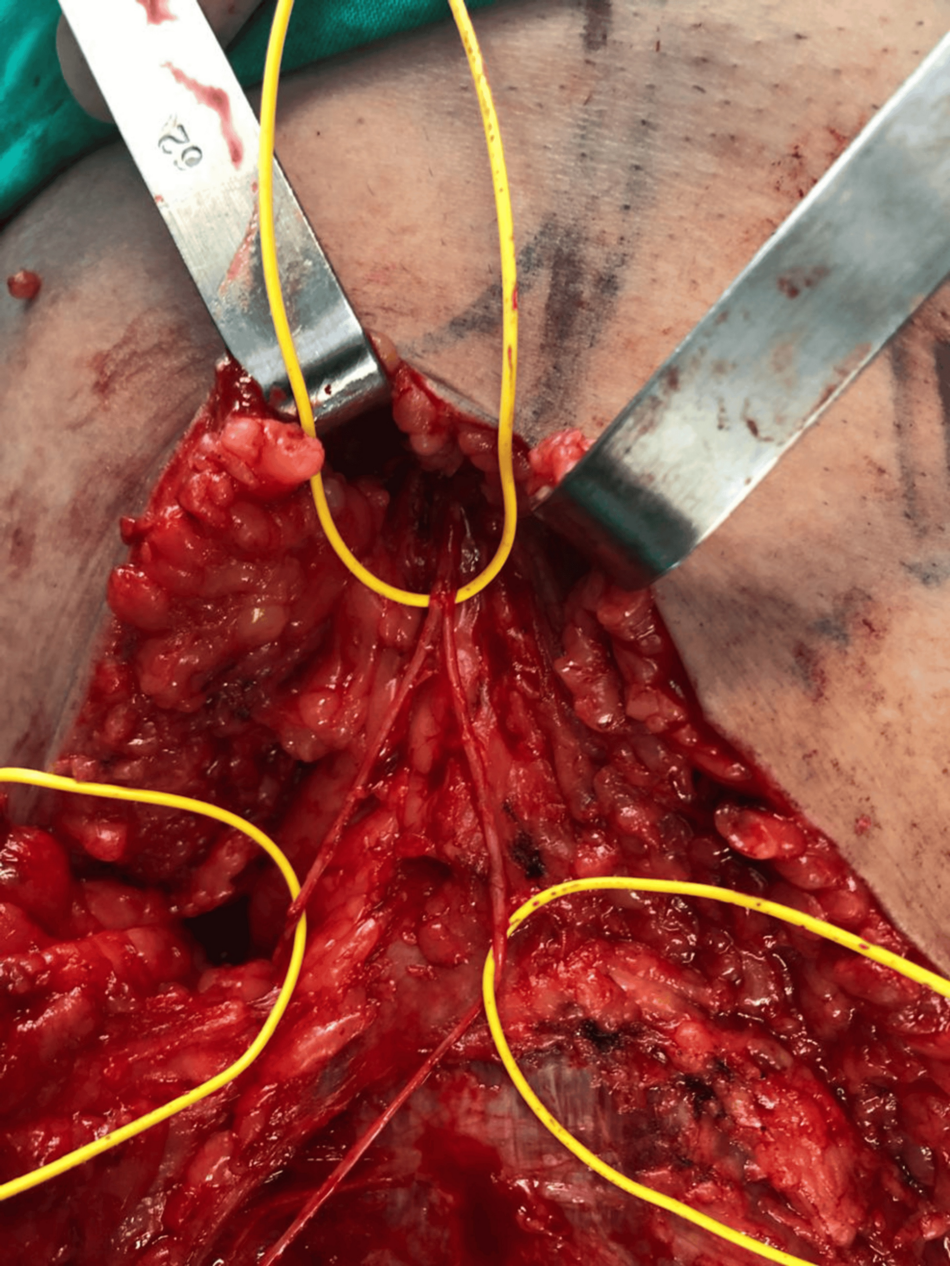
Identification of the lateral cutaneous femoral nerve.

The incision was first made along the flap’s medial border and continued into the skin and subcutaneous tissue until it reached the deep fascia. The musculocutaneous and septocutaneous perforators supplying the flap were then identified along with the lateral femoral cutaneous nerve, using subfascial dissection (Figures 2, 3).

**Figure 4:**
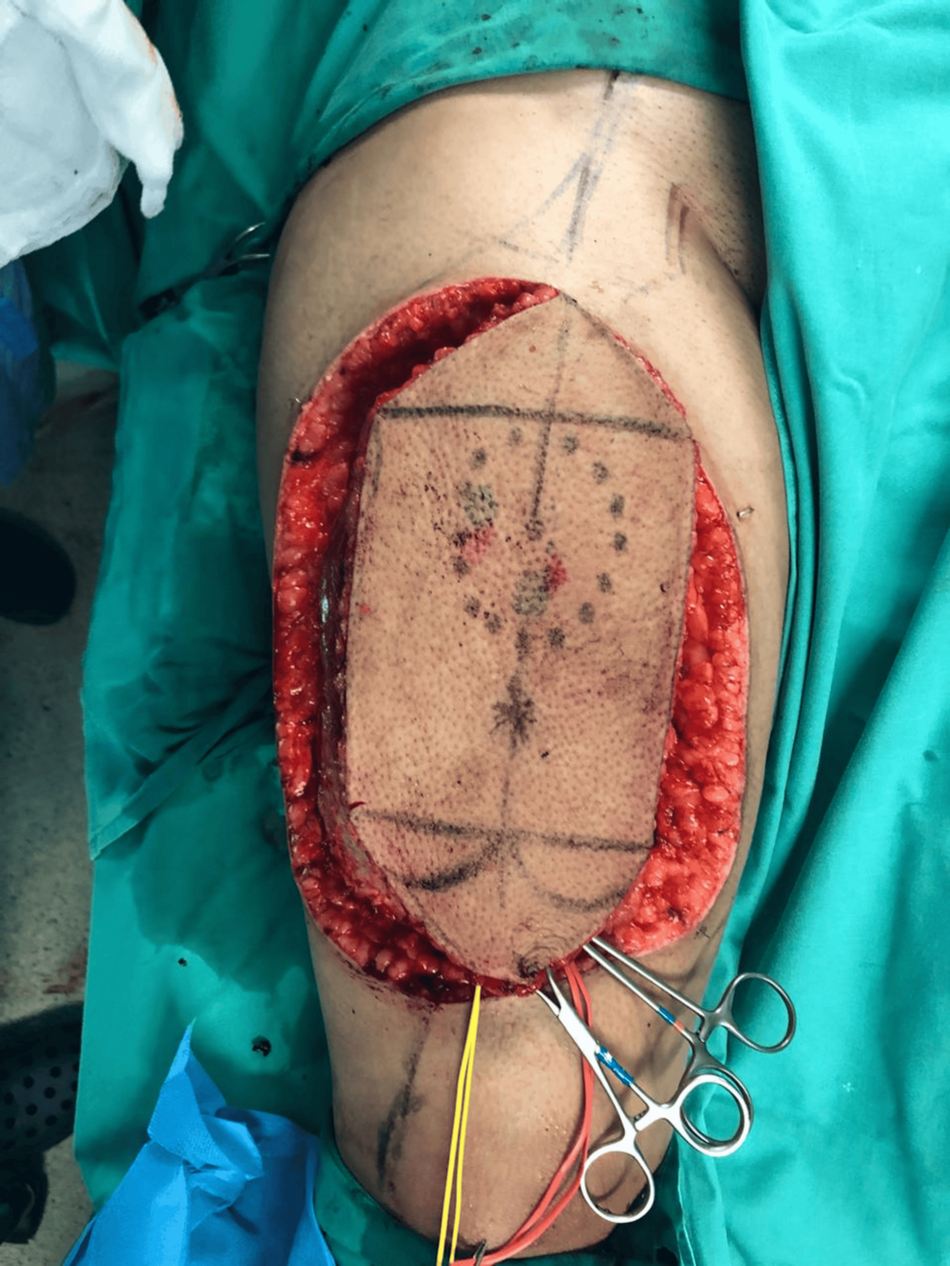
Mobilisation of the flap.

Following the identification of an appropriate perforator, the lateral circumflex femoral artery’s descending branch was carefully dissected proximally. To get enough length for transfer, the vascular pedicle was skeletonised. Careful haemostasis and perforator integrity maintenance were upheld during this phase. After that, the flap was harvested from distal to proximal while the vascular pedicle could still be seen (Figure 4).

A tunnel was then made towards the pubic symphysis in order to pass the flap along with its vascularisation and innervation to the recipient site underneath. The neophallus was created by tubularizing the extracted flap, while flap thinning was necessary in order to achieve the best aesthetic outcome. More precisely, this thinning was achieved by removing the deeper layer of the subcutaneous tissue, which is made up of bigger fat lobules. The principle that the perforator extends perpendicular to the subdermal plexus was used to accomplish debulking.

The shaft was formed by rolling the skin paddle longitudinally, and interrupted absorbable sutures were used to approximate the edges. The patient was able to maintain normal urine function without urethral lengthening. Prior to flap fixation, neurorrhaphy was done between the lateral femoral cutaneous nerve inside the flap and the dorsal nerve of the clitoris to aid in sensory recovery. The nerves were coapted end-to-end with fine micro-sutures under magnification.

**Figure 5:**
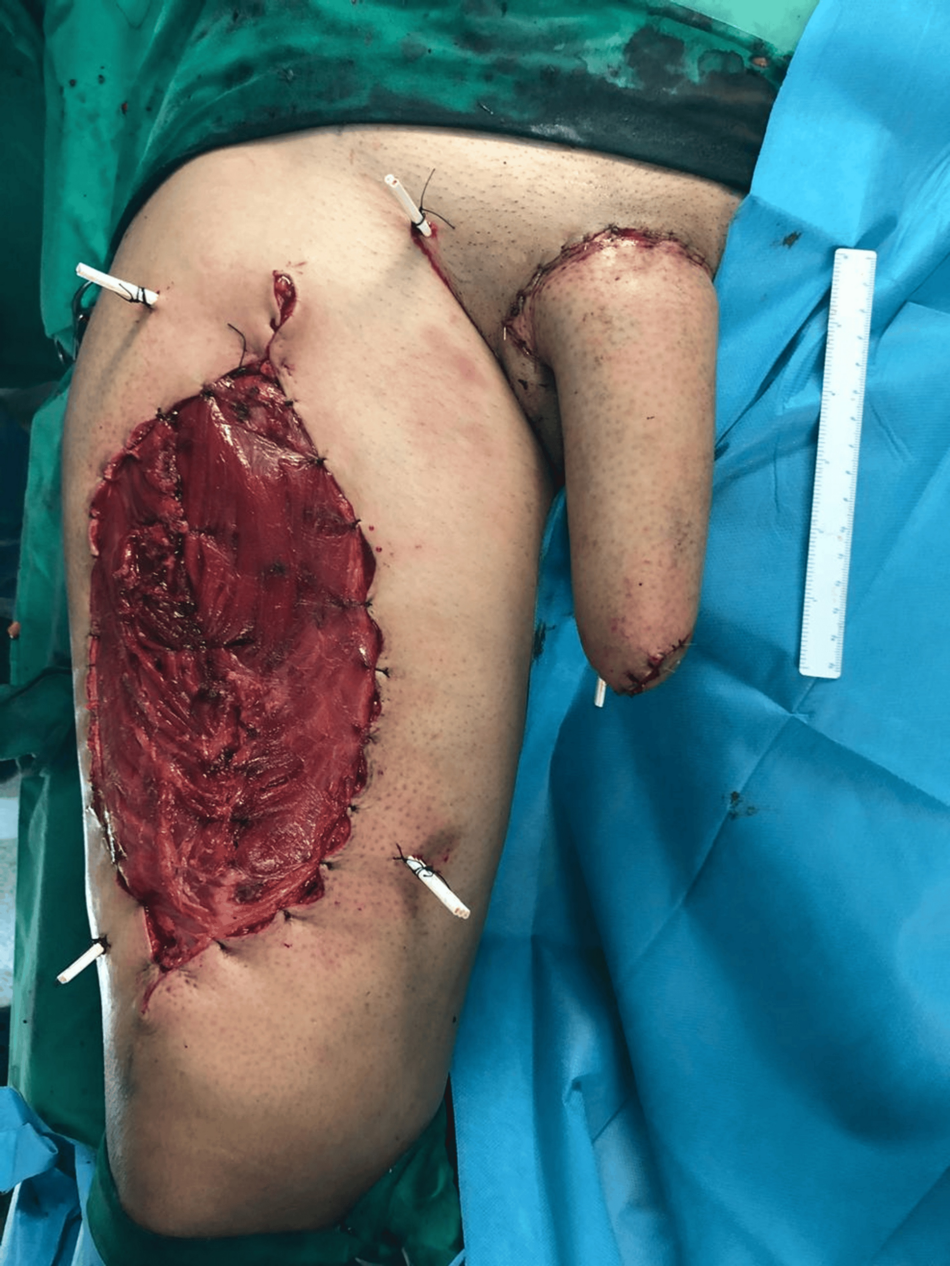
Preparation of the donor site for the Integra Dermal Regeneration Template (Integra LifeSciences, Princeton, NJ, USA).

For stable fixation and proper alignment, the neophallus was positioned and secured to the pubic area. The donor location was assessed and sealed with the Integra Dermal Regeneration Template (Integra LifeSciences, Princeton, NJ, USA) after the flap was harvested, enabling the best possible wound bed preparation while the edges were sutured, minimising in this way the depth of the wound from the skin level. Finally, closed suction drains were installed (Figure 5).

The neophallus showed good shape and sufficient perfusion at the end of the procedure. Microvascular anastomosis was not required since the flap maintained good vascularisation through its pedicle. There was no sign of flap compromise or wound-related problems during the postoperative period. In order to achieve complete and stable coverage, a split-thickness skin graft taken from the contralateral thigh was placed on the donor site in a second stage. For routine flap viability monitoring, the patient was moved to the postoperative unit. Anticoagulants, colloid fluids, antibiotic coverage, and continuous local thermal therapy were administered for five days. During follow-up, there were no indications of infection, graft loss, or delayed wound healing at either the recipient or donor sites. The patient was discharged from the hospital on the fifth postoperative day without any complications.

**Figure 6:**
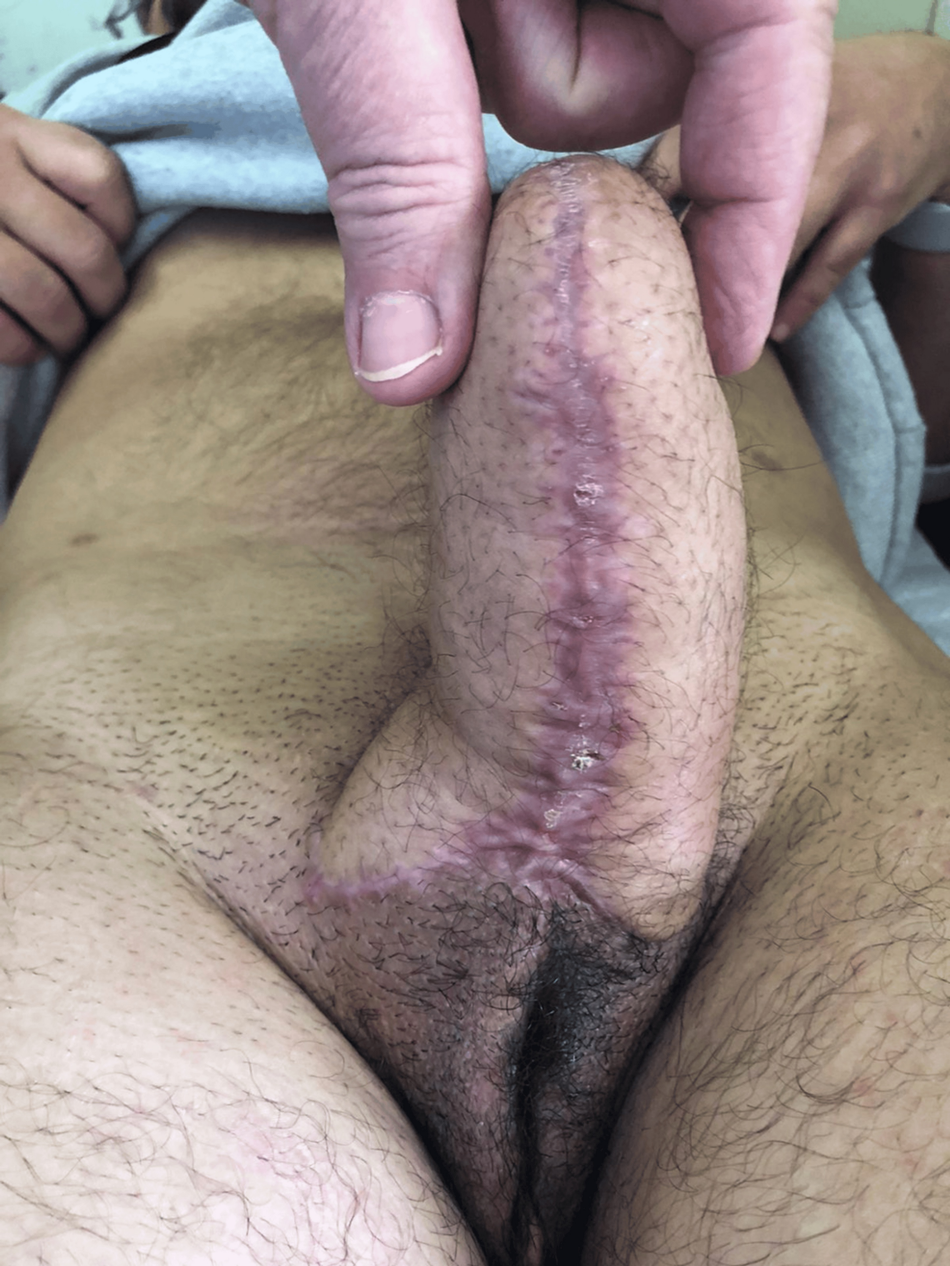
Postoperative neophallus.

The patient showed stable wound healing at both the donor and recipient sites at the one-month postoperative follow-up. There was no sign of wound dehiscence, infection, or partial flap loss, and the neophallus continued to be well perfused (Figure 6).

**Figure 7:**
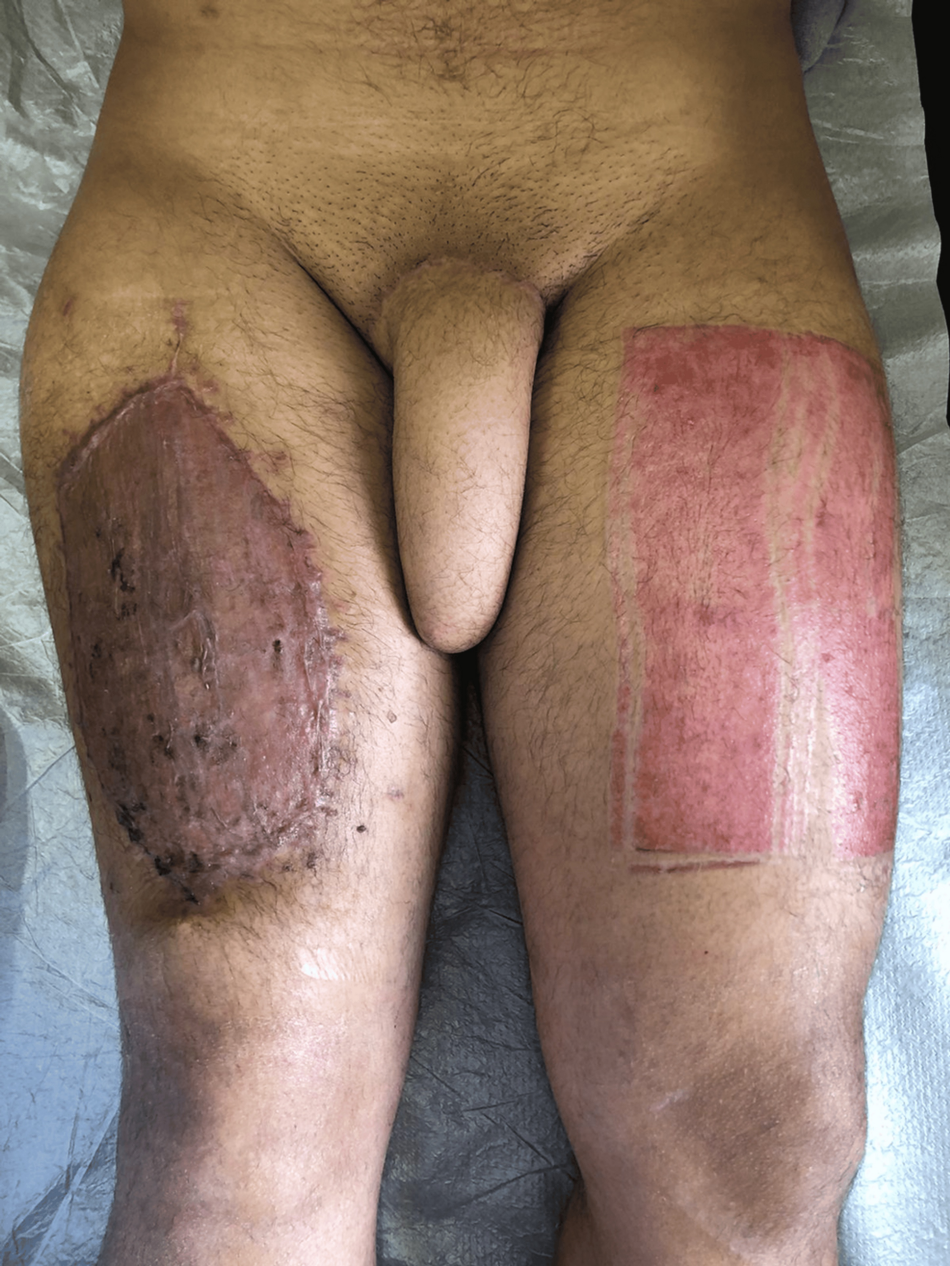
Postoperative wound healing.

The donor site demonstrated full graft take and good integration after being treated with a dermal regeneration template and split-thickness skin grafting (Figure 7).

Early indications of sensory improvement were observed, and the patient did not report any severe discomfort. At this point, both functional and aesthetic results were deemed satisfactory.

## Discussion

One of the most technically challenging reconstructive surgical operations in gender-affirming surgery is still phalloplasty, which necessitates striking a balance between functional results, donor-site morbidity reduction, and cosmetic appeal. With the pool of patients varying from transgender men and gender affirmation to penile reconstruction on cisgender men, the existing knowledge has to be challenged and the alternative techniques thoroughly studied in order to establish the most suitable choice for every case. Numerous methods have been reported, but because of its thin, flexible skin and dependable vascular structure, the radial forearm free flap is typically regarded as the most well-established choice. However, this method is linked to serious donor-site morbidity, including noticeable scarring and the possibility of skin grafting, which has led to a rise in interest in alternative reconstructive techniques. The pedicled ALT flap was selected for our patient, bearing in mind time efficiency, due to its lack of arterial microsurgical anastomoses, robust blood supply, but also their preferences regarding the scar site and the total length of the neophallus. Both free and pedicled transfers are made possible by the flap's lengthy vascular pedicle and large skin paddle. The pedicled version does not need microvascular anastomosis, which shortens the operating time and almost avoids major complications such as microvascular thrombosis. Those were the main reasons this method was chosen over the radial forearm flap, which, however, remains the gold standard of phalloplasty [6,9,13-15].

Urethral reconstruction, which is linked to high rates of complications, especially urethral fistula and stricture formation, is one of the biggest hurdles in phalloplasty. Although reported complication rates in the literature vary greatly, they continue to be a significant contributor to secondary procedures and morbidity. In this situation, maintaining the urethra intact after the metoidioplasty procedure he had previously undergone offers a straightforward and possibly safer option for patients who are carefully chosen. The main goal of neophallus creation is accomplished while the danger of urethral issues is successfully reduced by avoiding urethral extension. This strategy might be especially appropriate for patients whose primary concern is reducing surgical risk or for whom standing micturition is not a priority [16,17].

Restoring feeling is another crucial factor in phalloplasty. Both functional results and patient satisfaction are greatly impacted by sensory recovery. In order to increase the neophallus’s sensory potential, neurorrhaphy between the lateral femoral cutaneous nerve and the dorsal nerve of the clitoris was carried out. Although the degree and quality of sensation may differ, prior research has shown that nerve coaptation in flap-based phalloplasty can lead to significant sensory recovery over time. A significant step toward enhancing long-term functional results is the addition of a sensate flap in conjunction with nerve coaptation [18,19].

Significant benefits in quality of life, especially in the areas of mental health, body image, and social functioning, have been repeatedly linked to phalloplasty and gender-affirming surgical treatments. Meier and Papadopulos' 2021 systematic analysis of quality of life following gender reassignment surgery showed that surgical intervention improves psychological well-being, increases body and gender identity satisfaction, and improves overall life satisfaction [20]. Additionally, studies that concentrate on phalloplasty in particular reveal high levels of patient satisfaction with changes in everyday functioning, sexual function, and self-esteem, all of which have a significant positive impact on quality of life. However, it's crucial to remember that, even with these advancements, quality of life can still be worse than in the general population, underscoring the necessity of continuing interdisciplinary assistance [20,21].

The ALT flap has drawbacks despite its benefits. Compared to forearm-based flaps, the flap is frequently larger, which could lead to a bulkier neophallus and sometimes require further debulking surgeries to improve form. Furthermore, the skin can be hairy, which could make the patient's everyday life difficult [22-24].

## Conclusions

To conclude, the pedicled ALT flap deserves to be taken into consideration alongside the radial forearm free flap, abdominal flaps, and other thigh-based flaps when perioperative planning is made. In certain patients, a pedicled ALT flap that preserves the native urethra may be a successful reconstructive choice because it avoids microvascular anastomosis, reduces surgical complexity, and lowers the risk of urethral issues. Additionally, nerve coaptation may improve sensory results, which would increase patient satisfaction in general.

However, this report's conclusions on long-term results and generalizability are still constrained by its single-case design. To properly define this technique's place among the range of phalloplasty alternatives, more research with larger patient cohorts and longer follow-up is required. Last but not least, such operations have been linked to significant gains in quality of life, including improved psychological well-being, body image, and social integration, in addition to these medical advantages, and that is why it is a crucial component of a multistructured transgender treatment program. Thus, phalloplasty contributes significantly to long-term quality of life and overall patient satisfaction beyond anatomical repair, but the results must be confirmed in prospective research, including preoperative assessments.
